# Charcot osteoarthropathy in type 2 diabetes persons presenting to specialist diabetes clinic at a tertiary care hospital

**DOI:** 10.1186/s12902-015-0023-4

**Published:** 2015-06-12

**Authors:** Bilal Bin Younis, Adeela Shahid, Rozina Arshad, Saima Khurshid, Junaid Masood

**Affiliations:** Department of Endocrinology Medicine, Shalamar Medical and Dental College Lahore, Shalamar Link Road Lahore, Postal address: 2-J Gulberg III Lahore, Pakistan; Department of Physiology, Shalamar Medical and Dental College, Lahore, Pakistan; Department of Physiology and Cell Biology, University of Health Sciences, Lahore, Pakistan

**Keywords:** Charcot foot, Diabetes, Deformity, Neuropathy, Foot care

## Abstract

**Background:**

Charcot osteoarthropathy or charcot foot is a rare, chronic, non-communicable condition of bones and joints which may results into severe deformity and more prone to develop ulcers possibly leading to amputation. The purpose of this study was to determine the prevalence of Charcot osteoarthropathy and its association with age, BMI, gender, duration of diabetes, HBA1c and peripheral neuropathy.

**Methods:**

A total of 1931 subjects with type 2 diabetes having mean age 50.72 ± 10.66 years presenting in a specialist diabetes clinic at shalamar hospital, Lahore, Pakistan were enrolled. The diagnosis of Charcot osteoarthropathy was made by examination of both dorsal and plantar surfaces of foot for swelling, erythema, increase in temperature and any musculoskeletal deformity which was later confirmed by radiographs. Assessment of neuropathy was carried out by checking the sense of pressure, joint position and vibration. BMI (Body Mass Index), fasting blood glucose (FBG) and HbA1C were determined.

**Results:**

In all subjects including male 704 (36.45 %) and female 1227 (63.55 %), 0.4 % subjects had charcot deformity, while 0.2 %, 0.15 % and 0.05 % subjects having right, left and bilateral deformity respectively. Bilaterally symmetrical neuropathy was diagnosed in 25.4 % in subjects. There was a significant association (p < 0.05) of deformity with duration of diabetes, HbA1C and neuropathy, however no significant association (p > 0.05) was found with age, BMI, weight, height and gender.

**Conclusion:**

There is a need to have a special care of persons with diabetes regarding blood glucose control and development of peripheral neuropathy. Early identification and management of risk factors may prevent the occurrence of charcot deformity. Patients must be educated about the foot care.

## Background

Diabetes is a chronic condition associated with the number of complications such as peripheral artery disease, foot ulcerations, peripheral neuropathy and charcot osteoarthropathy. These complications are expected to increase in the prevalence [1] as the worldwide prevalence of diabetes for all age groups is expected to be 4.4 % by 2030 which was approximately 2.8 % in the year 2000 [2]. In Pakistan, according to the International Diabetes Federation (IDF) the prevalence of diabetes is quite high, about 6.7 million of the population has diabetes. By the year 2035, which is likely to increase to 12.8 million [3].

Charcot osteoarthropathy is a most overlooked serious limb-threatening complication of diabetes mellitus. Charcot foot was first described in 1883, and it was associated to diabetes mellitus in 1936 [4]. It is a chronic progressive noncommunicable disease of the joints and bones which may involve single or multiple bones or joints. It is characterized by painless swelling in the feet and ankles along with damage in bones and joints. It can lead to deformity of bone and joint due to underlying neuropathy, trauma or any disturbance in bone metabolism [5]. The classic deformity associated with Charcot foot is “rocker bottom deformity” which is a collapse of midfoot [6]. Charcot osteoarthropathy occurs mostly in persons with diabetes with severe peripheral neuropathy [7, 8]. The prevalence of Charcot osteoarthropathy is reported to be 0.08 % to 13 % in individual with diabetes and high risk patients respectively [7].

Foot complications are now considered to be the most common cause of hospital admission and lower limb amputation in subjects with diabetes. The diagnosis of charcot foot is not easy and is problematic even for the very experienced practitioners. Its diagnosis in early stages is extremely difficult and many cases of charcot foot are misdiagnosed. The actual prevalence may be higher due to delay in diagnosis or misdiagnosis [9].

X-rays plays a major role in diagnosis of charcot foot and helps in distinguishing it from osteomyelitis. Other imaging techniques such as CT Scan, MRI and PET scan can also be used to diagnose charcot foot [4].

The aim of the study was to determine the prevalence of Charcot foot and its association with age, gender, duration of diabetes, control of blood sugar level and neuropathy.

## Materials and methods

### Data collection

It was a cross sectional study including a total of 1931 (mean age 50.72 ± 10.66) type 2 diabetes persons including both male and female presenting in Sakina Institute of Diabetes & Endocrine Research (SiDER) which is a specialist diabetes clinic at Shalamar Institute of Health Sciences, Lahore, Pakistan, over period of one year from Sept 2012 to August 2013. The cognitive impaired subjects were excluded from the study. The demographic information (name, age, sex, address, education, duration of diabetes) was collected from customized designed hospital based software. Data entry was done by a trained doctor proficient in using the software. The maintenance of data and software was done by hospital IT department. The Ethical Review Committee of Shalamar Institute of Health Sciences reviewed and approved the study. The diabetic condition was confirmed by review of medical record, previous laboratory tests. The patients having fasting blood glucose ≥126 mg/dl were considered as diabetic. The written informed consent was taken after explaining the nature of the study to the patients.

### Examination

Complete general physical and systemic examination was carried out. Examination of the arterial pulse in dorsalis pedis and posterior tibial arteries were palpated and compared bilaterally.

### Charcot osteoarthropathy

Physical examination of the lower limb was carried out; diagnosis of Charcot osteoarthropathy of foot was primarily based on history and clinical examination. Both dorsal and plantar surfaces of the foot were examined for swelling, erythema, increase in temperature and any musculoskeletal deformity which were later on confirmed by X-rays.

### Body height and weight

The standard procedure was followed to measure body weight and height. Body weight was measured using a digital scale whereas a wall-mounted stadiometer was used to measure body height. Then, BMI of each subject was calculated.

### Assessment for neuropathy

The examination of the patients was done for the presence of neuropathy, the sense of pressure, joint position and vibration were checked with the standard method and each test was performed three times. 128 Hz tuning fork was used to check the sense of vibration by taking halluces as standard point, if the subject felt the vibration it was considered as positive and if the subject failed to perceive the vibration sensation while the person examining still felt, test was recorded as negative. The vibration sense was considered as impaired if two out of three times the subject was unable to perceive the vibration.

The sense of position was checked according to the standard clinical practice which is dorsal flexion and plantar flexion at interphalangeal joint. The interphalangeal joint of the big toe was flexed for 10^0^ in upward and downward direction and the subject was asked to tell the position of the joint.

The loss of proper proprioception is documented if the patient could not identify the right direction of the big toe. The test was performed three times, if the patient could not identify the position two out of three times the test was considered impaired.

Semmes-Weinstein (SW) monofilament (10gm) was used to check the pressure sensation of the persons at four sites namely 1st, 3rd, and 5th metatarsal heads and plantar surface of distal hallux as recommended by Boulton A J et al. [10]. The pressure was applied with 10 g monofilament perpendicular to the above mentioned sites for 2 seconds at least and the persons were asked to nod their head or say ‘yes’ when they felt the monofilament. The test was reported as abnormal if patient is unable to perceive the sensation two out of three times. Abnormal test is considered as impairment of sensation.

### Analytical determinations

The sample of 4 ml of venous blood was collected from the cubital vein after overnight fasting of 12 h. All parameters and were recorded in duplicate. Standard procedures were followed to determine the parameters. The commercial reagent kit (ACON Laboratories, San Diego, California, USA) was used to measure fasting blood glucose levels by the glucose oxidase method. HbA1-c was estimated by high affinity liquid chromatography with a D-SI Glycomat (Provalis Diagnostics, Deeside, UK).

### Statistical analysis

SPSS (Statistical Package for Social Sciences) version 17.0 [SPSS, Inc. Chicago, IL, USA] was used to enter and analyze data. The Quantitative variables were expressed as mean ± standard deviation (S.D), whereas qualitative data was presented by frequency and percentages.

The Kruskal Wallis test was applied to determine association of age, BMI, HbA1c, duration of diabetes for deformity. A p-value of < 0.05 was considered statistically significant.

## Results

### Characteristics of study population

A total of 1931 type 2 persons with diabetes, including male 704 (36.45 %) and female 1227 (63.55 %) were recruited. The mean age was 50 ± 10.66 years with the duration of diabetes 7.16 ± 6.28 years. The sample population has mean BMI of 23.26 ± 4.59 kg/m^2^. The characteristics of study population are given in Table 1.

**Table 1:** Characteristics of study population

Parameters	
Total Sample population (n)	1931
Age (years)	50.72 ± 10.66
Gender	
Male (%)	704(36.45 %)
Female (%)	1227(63.55 %)
Duration of Diabetes (years)	7.16 ± 6.28
Body weight (Kg)	73.80 ± 15.1
Body Height (cm)	158 ± 10.7
BMI (kg/m^2^)	23.26 ± 4.59
HbA1C (%)	9.61 ± 2.19

Data are expressed as mean ± SD and frequencies

### Charcot osteoarthropathy

A total of 1931 subjects were examined for charcot deformity. In 99.6 % of the subjects, no deformity was found while 0.4 % of the subjects had the deformity. When these subjects were further evaluated, 0.15 % of the all subjects and 37.5 % of affected subjects had the charcot deformity of right foot while 0.2 % of all subjects and 50 % of affected individuals had the deformity of left foot. In 0.05 % among whole sample population and 12.5 % of reported charcot had the bilateral deformity as shown in Table 2.

**Table 2:** Charcot osteoarthropathy in persons with diabetes

Characteristics	Number	Percentage
Total sample population	1931	100 %
Deformity absent	1923	99.6 %
Deformity present	8	0.4 %
Deformity of right foot	3	0.15 %
Deformity of left foot	4	0.2 %
Bilateral deformity	1	0.05 %

Data are expressed as frequencies and percentage

The significant association of charcot osteoarthropathy was observed with duration of diabetes (*χ*^2^ = 4.37, p = 0.036), neuropathy (*χ*^2^ = 4.017, p = 0.045) and HbA1c (*χ*^2^ = 9.081, p = 0.003) respectively with their mean rank shown in the Table 3, whereas no association was found with BMI (*χ*^2^ = 0.05, p = 0.036), gender (*χ*^2^ = 2.35,1 p = 0.125), height (*χ*^2^ = 1.345, p = 0.246), weight (*χ*^2^ = 0.001, p = 0.971,) and age (*χ*^2^ = 2.11, p = 0.146). The data was stratified into different age groups and it was found that age group of 60-79 years shared the 50 % (n = 4) burden of deformity found as given in Table 4.

**Table 3:** Mean Rank of Charcot Osteoarthropathy in people with diabetes

	With Charcot	Without Charcot	*χ*2/ P-value
	osteoarthropathy	osteoarthropathy	
	(n = 8)	(n = 1923)	
Age	964.81	1251.12	2.11/0.146
Body weight	966.03	958.75	0.001/0.971
Height	965.05	1194.00	1.34/0.246
BMI	966.23	911.62	0.07/0.782
Duration of diabetes	960.79	1371.88	4.3/0.036*
HbA1C	405.03	656.75	9.08/0.003*

kruskul-Wallis test. p* < 0.05 was considered statistically significant

**Table 4:** Prevalence of Charcot osteoarthropathy in different age group

Age groups	No deformity		Deformity	
(years)	n = 1923		n = 8	
	n	%	n	%
<35	92	4.7	0	0
35 - 44	402	20.8	2	0.1
45 - 59	974	50.4	2	0.1
60 - 79	441	22.8	4	0.2
>79	14	0.72	0	0

### Neuropathy

All the subjects were examined for neuropathy. It was found that bilaterally symmetrical peripheral neuropathy was present in 25.6 % in subjects.

## Discussion

Presently, the most common cause of osteoarthropathy is diabetes over the world [11–15] and prevalence of diabetes is increasing worldwide [3]. The prevalence of charcot osteoarthropathy was found to be 0.4 % in subjects with diabetes in the current study, however previous data reported 0.08-7.5 % prevalence [16].

Charcot osteoarthropathy is present in unilateral limb in most cases but it can be present in bilateral limbs [17, 18]. Bilateral deformity was observed in 12.5 % of the reported cases of charcot osteoarthropathy in the present study, which is similar to the prevalence of 9 % and 12 % of the persons with diabetes in previous studies [19, 20].

Neuropathy was observed in 25.6 % of the population studied in the current study, whereas 28 % of persons with neuropathy was reported in european diabetes centre study of complication [21] while a study reported 42.1 % of the persons had sensory neuropathy and 2 % of them had Charcot osteoarthropathy [22]. However, the concomitant presence of deformity and peripheral neuropathy significantly increase the chances of skin ulceration and lower limb amputation [18].

There was no gender related differences observed in relation to charcot osteoarthropathy in the present study. Whereas a study has indicated that men and women are equally affected with charcot osteoarthropathy [23]. Stuck and colleagues suggested that the obesity is also a predisposing factor for charcot osteoarthropathy in european population [24]. In contrast to this, we failed to find any significant association of charcot osteoarthropathy with BMI. This difference might be due to regional, ethnic or due to difference in sample size and statistical methods used.

Deformity is associated with duration of diabetes and HbA1C in the current study. Likewise, Charcot osteoarthropathy has been associated with longstanding duration of diabetes and neuropathy in previous studies [24, 25]. As far as age is concerned, the highest prevalence of deformity has been reported in age group of more than 60 years [24]. Our study also reported more prevalence in more than 60 years old individuals.

It has been observed that neuropathy in the presence of deformity or limited joint mobility results in 12.1 times higher risk of foot problems, whereas patients who had ulcer or amputation done in the past have a 36.4 times higher risk for developing another ulcer [26], it might be linked to superimposed infection because such persons are more likely to develop recurrent ulcers.

This condition is more focused now because it is not only the cause of chronic progressive deformity but also intensifies the likelihood of major amputation [27].

## Conclusion

Although the prevalence of charcot osteoarthropathy in this sample population is relatively low, but it has potential risks such as permanent disability, amputation and recurrent ulcers. It was found that neuropathy, HbA1c and duration of diabetes are predisposing risk factors for charcot osteoarthropathy while age, gender and BMI are not significant risk factors.

The earlier diagnosis, timely appropriate treatment may help in avoiding severe, irreversible disability and deformity, which can be done with the tool of patient education and conducting seminar to increase awareness.
